# Massively calcified aneurysm of the anterior communicating artery: an unsuccessful clipping attempt followed by successful pCONus2-assisted coil occlusion

**DOI:** 10.1093/jscr/rjab107

**Published:** 2021-04-19

**Authors:** Amer Haj, Nils Ole Schmidt, Christina Wendl, Karl-Michael Schebesch

**Affiliations:** Department of Neurosurgery, University Medical Center Regensburg, Regensburg, Germany; Department of Neurosurgery, University Medical Center Regensburg, Regensburg, Germany; Department of Neuroradiology, University Medical Center Regensburg, Regensburg, Germany; Department of Neurosurgery, University Medical Center Regensburg, Regensburg, Germany

## Abstract

We report the unique case of a 67-year-old female patient with an incidentally discovered massively calcified aneurysm of the anterior communicating artery. After interdisciplinary discussion, we decided to attempt clipping the aneurysm because interventional therapy did not seem feasible and anchoring a stent would also have been difficult because of the morphology of the aneurysm. The clipping attempt was unsuccessful because of the aneurysm’s massive calcification with strong adherence to the sphenoid planum. The aneurysm was then successfully occluded using pCONus 2-assisted coiling. We illustrate this and consecutive procedures, and we reviewed the literature on aneurysm calcification.

## INTRODUCTION

To prevent subarachnoid hemorrhage, patients with aneurysm usually undergo prophylactic treatment. Microsurgical clipping was the primary treatment prior to the introduction of endovascular procedures that have significantly increased after the international subarachnoid aneurysm trial [1] and the international study of unruptured intracranial aneurysms [2]. Interdisciplinary discussion of each individual patient has been widely demanded to evaluate the best possible treatment.

We present a unique case of a large multi-lobulated anterior communicating artery (Acom) aneurysm with a wide neck, extreme calcification and inseparable adherence to the anterior skull base, in which the clipping attempt was unsuccessful.

## CASE REPORT

A 67-year-old female patient without any significant past medical history was referred to our neurovascular center for treatment of an Acom aneurysm that had been incidentally discovered during magnetic resonance imaging (MRI).

**Figure 1:**
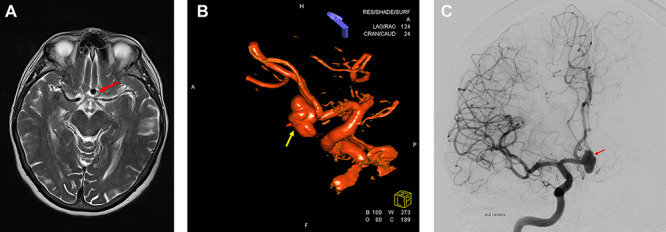
Preoperative neuroimaging. (**A**) MRI T2-weighted sequence shows an Acom aneurysm without thrombosis. (**B**, **C**) Digital subtraction angiography shows a multi-lobulated Acom aneurysm approximately sized 13.0 × 7.5 mm. (B) 3D Digital subtraction.

MRI and digitally subtracted angiography showed a large multi-lobulated Acom aneurysm approximately sized 13.0 × 7.5 mm (Fig. 1A–C).

Endovascular coil occlusion was not considered first-choice treatment because of expected difficulties in properly accessing the aneurysm and in anchoring a stent because of the wide neck and the sharp angle between the aneurysm and the adjacent vessels (anterior cerebral arteries A1 and A2). Consequently, clipping was recommended.

### Surgery

**Figure 2:**
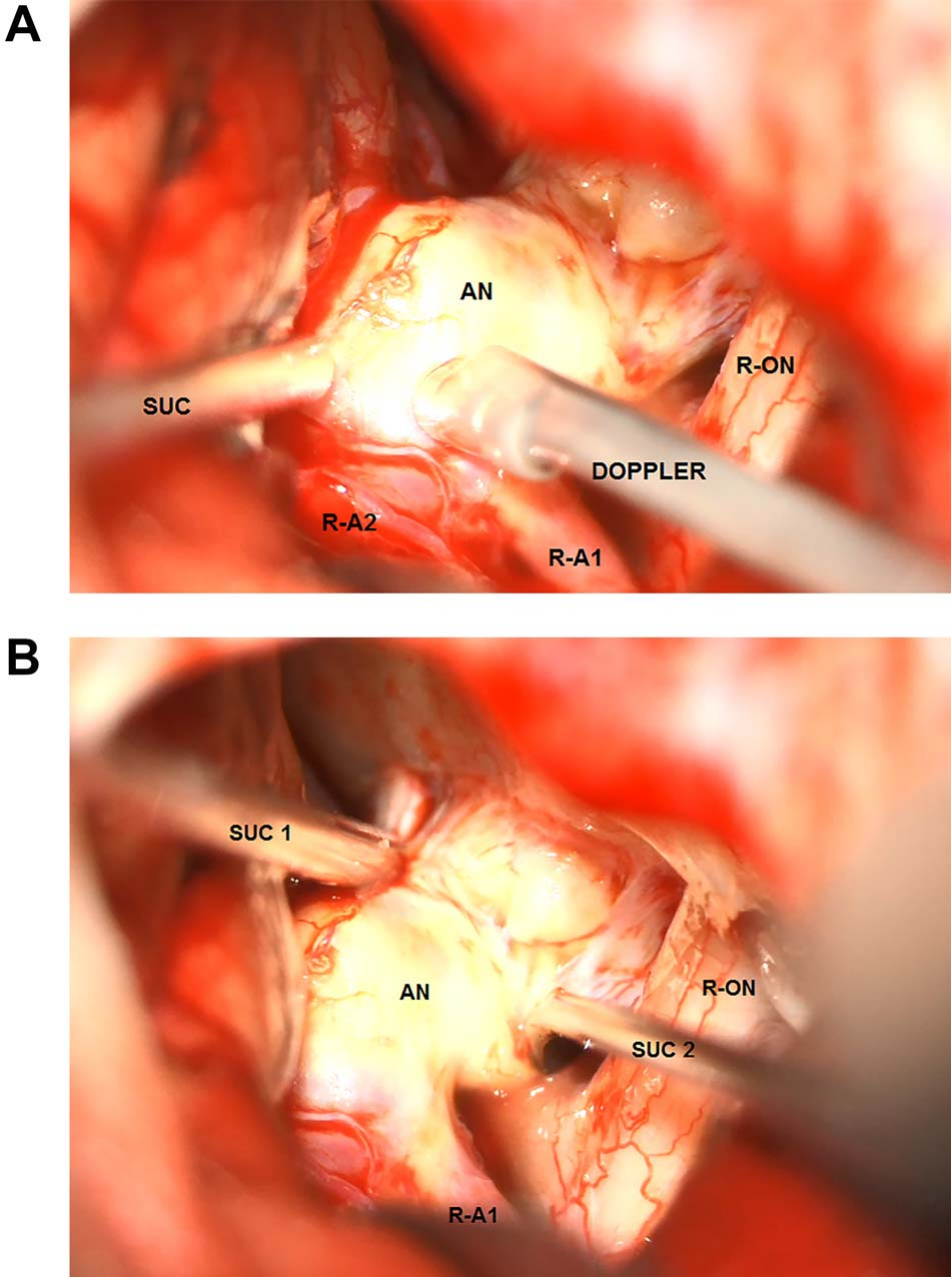
Intraoperative microscopic transsylvian view of the aneurysm. (**A**) Intraoperative microscopic transsylvian view of the aneurysm after the failed attempt to compress the calcified aneurysm with two suction devices (AN = aneurysm, R-ON = right optic nerve, SUC = suction, and R-A1 = right anterior cerebral artery). (**B**) Intraoperative microscopic transsylvian view of the aneurysm showing the assessment of the flow inside the aneurysm by means of micro-Doppler examination after temporary clipping of the right A1 segment. (AN = aneurysm, R-ON = right optic nerve, SUC = suction, DOPPLER = micro-Doppler, R-A1 and R-A2 = right anterior cerebral arteries).

After pterional craniotomy on the right side, the anterior vasculature, optic nerves and chiasm were dissected, and all perforating vessels were preserved. The anteriorly directed part of the aneurysm was massively calcified, even ossified and adhered to the skull base at the level of the tuberculum sellae (see Fig. 2A and B). Thus, separating the aneurysm from the anterior skull base was not possible. The right A1 segment was temporarily clipped. The flow inside the aneurysm was assessed by means of micro-Doppler examination that showed a preserved intensive perfusion signal from the left side (Fig. 2B). The aneurysm was further dissected, but the right A2 segment could not be separated from the bulky, rigid aneurysm sack. Compression of the aneurysm was also not possible, and no clip could be positioned because of massive calcification of the aneurysm wall (Fig. 2A). After circumferential dissection and consecutive identification of all vessels as well as repeated attempts to compress the aneurysm and to restrain the flow, surgery was finally discontinued because of the increasing risk of aneurysm rupture in the case of further manipulation.

After further discussion with the interventional neuroradiologists, endovascular treatment with pCONus2-assisted coil occlusion was recommended, to which the patient agreed.

**Figure 3:**
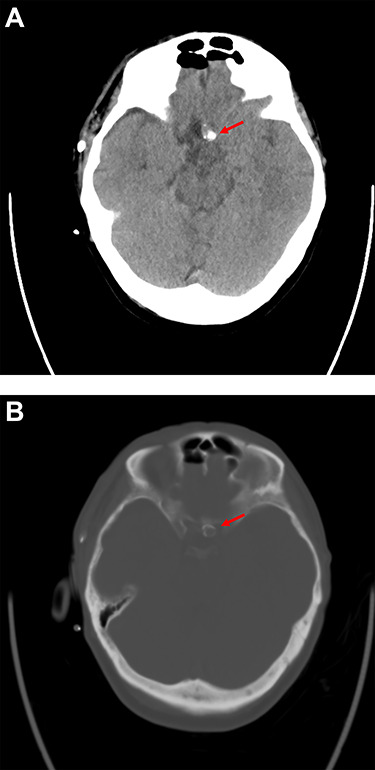
Postoperative computer tomography (**A**. soft tissue window, **B**. bone window) shows calcification of the aneurysm neck and dome with adherence to the skull base at the level of the tuberculum sellae.

Postoperative cranial computed tomography (CT) showed massive calcification of the aneurysm neck and dome with adherence to the skull base at the level of the tuberculum sellae; the CT results corresponded well to the intraoperative findings (Fig. 3A and B).

### Endovascular treatment

**Figure 4:**
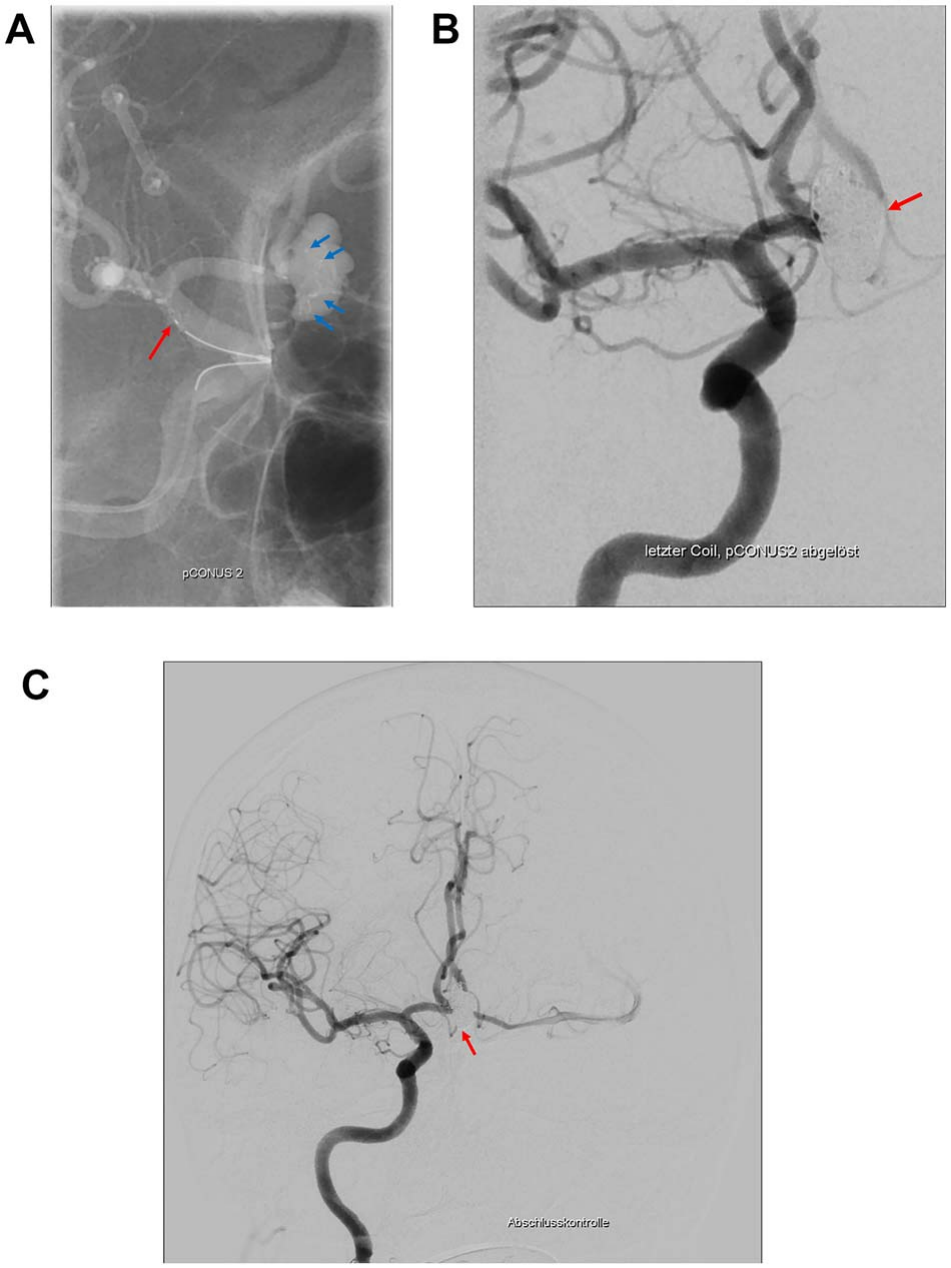
The pCONus2 device for the treatment of a calcified aneurysm of the Acom. (**A**) Digital subtraction angiography showing the placement of the pCONus2 bifurcation stent with its petals being located at the level of the aneurysm neck and the proximal stent ending at the distal segment of the internal carotid artery. (**B**) The implantation of several coils with a second micro_catheter navigated through the already opened pCONus. (**C**) Postprocedural digital subtraction angiography showing the completely occluded aneurysm with preservation of the Acom.

Under general anesthesia, a six French guiding catheter (Envoy, Codman Neuro, Raynham and USA) was positioned in the cervical segment of the right internal carotid artery. A Prowler Select 21 micro catheter (Cordis, Santa Clara and USA) was navigated into the aneurysm followed by the uncomplicated implantation of an HPC-coated pCONus2 bifurcation stent (phenox, Bochum and Germany; Fig. 4A). The petals of the device covered the wide neck of the aneurysm, enabling the complete occlusion of the aneurysm with coils (Fig. 4B). Final control showed complete occlusion of the aneurysm as well as patent perfusion of both anterior cerebral arteries (Fig. 4C). No embolic events were observed. Clinical outcome was excellent according to the glasgow outcome scale 5 and modified ranking scale 0. The patient was discharged and kept on dual antiplatelets (Clopidogrel and Aspirin) for 6 months and single antiplatelet (aspirin) thereafter for another 6 months.

## DISCUSSION

Most authors have agreed that, apart from calcification, patient age, the size and site of the aneurysm are also important variables that potentially affect surgical outcome [3–5], Bhatia *et al.* claimed that the presence of calcification alone is the sole marker of adverse outcome, which was associated with a 7.8 times higher risk in the case of aneurysm calcification. They also suggested that the size of an aneurysm does not adversely affect the surgical result; size just indirectly influences outcome because larger aneurysms probably show a higher degree of calcification [6]. The reason for potentially compromised outcome is that calcification renders the aneurysm neck stiff and breakable, which may cause tearing or cracking of the wall and local plaques to become loose during clipping. Furthermore, calcification may prevent correct positioning of the clip on the neck, thus hindering proper closure with consecutive residual perfusion as in our patient. Calcification may also result in slipping of the clip and subsequently in repeated clip adjustments and the occlusion of distal branches [3, 4, 6]. In our patient, the aneurysm was massively calcified, rather ossified and had become inseparable from the skull which made clipping extremely dangerous. Therefore, we decided to discontinue surgery and reconsider endovascular treatment as an alternative.

Wide-necked bifurcation aneurysms continue to present a challenge for interventionalists [7]. pCONus devices (Fig. 4) first introduced in 2014 for wide-necked bifurcation aneurysms-are novel devices using the ‘waffle-cone’ technique [8]. These implants are intra-saccular devices that do not affect blood flow because of a stent body structure with an articulated crown of pedals, which are gently pressed against the vessel wall. The pCONus2 device has additional pedals for improved coverage of the aneurysm neck and a shorter shaft to decrease coil prolapses into the parent vessel [9].

In their meta-analysis, Sorenson et al. found a technical success rate of 100% and a technical complication rate of 0% for pCONus devices. Perioperative morbidity and mortality rates were also low (7 and 0%, respectively; [10]).

Because of the aneurysm morphology, we decided to use the pCONus2-assisted coil device. Consequently, complete endovascular occlusion of the aneurysm was successful with excellent outcome.

## CONCLUSIONS

In selected patients with complex large to giant aneurysms we suggest performing a diagnostic computed tomography angiography which is a practical method for evaluating calcification, as larger aneurysms tend to be calcified. Since calcification can become an obstacle to surgery, anticipating calcification improves decision-making in choosing the ideal individualized treatment in a multimodality system, and therefore can improve outcome.

## References

[1] Molyneux AJ, Kerr RS, Yu LM, Clarke M, Sneade M, Yarnold JA, et al. International subarachnoid aneurysm trial (ISAT) of neurosurgical clipping versus endovascular coiling in 2143 patients with ruptured intracranial aneurysms: a randomised comparison of effects on survival, dependency, seizures, rebleeding, subgroups, and aneurysm occlusion. Lancet (London, England) 2005;366:809–17. 10.1016/S0140-6736(05)67214-5 16139655

[2] Wiebers DO, Whisnant JP, Huston J, 3rd Meissner I, Brown RD Jr, Piepgras DG, et al. Unruptured intracranial aneurysms: natural history, clinical outcome, and risks of surgical and endovascular treatment. Lancet (London, England) 2003;362: 103–10. 10.1016/s0140-6736(03)13860-3 12867109

[3] Grigorian AA, Marcovici A, Flamm ES. Intraoperative factors associated with surgical outcome in patients with unruptured cerebral aneurysms: the experience of a single surgeon. J Neurosurg 2003;99:452–7. 12959429 10.3171/jns.2003.99.3.0452

[4] Kizilkilic O, Huseynov E, Kandemirli SG, Kocer N, Islak C. Detection of wall and neck calcification of unruptured intracranial aneurysms with flat-detector computed tomography. Interventional neuroradiology: journal of peritherapeutic neuroradiology, surgical procedures and related neurosciences 2016;22:293–8. 10.1177/1591019915626591 PMC4984361 26842608

[5] Kasinathan S, Yamada Y, Cheikh A, Teranishi T, Kawase T, Kato Y. Prognostic factors influencing outcome in Unruptured anterior communicating artery aneurysm after microsurgical clipping. Asian journal of neurosurgery 2019;14:28–34. 30937004 10.4103/ajns.AJNS_198_18 PMC6417356

[6] Bhatia S, Sekula RF, Quigley MR, Williams R, Ku A. Role of calcification in the outcomes of treated, unruptured, intracerebral aneurysms. Acta Neurochir 2011;153:905–11. 21286763 10.1007/s00701-010-0846-8

[7] Pierot L, Cognard C, Spelle L, Moret J. Safety and efficacy of balloon Remodeling technique during endovascular treatment of intracranial aneurysms: critical review of the literature. Am J Neuroradiol 2012;33:12–5. 21349960 10.3174/ajnr.A2403 PMC7966153

[8] Horowitz M, Levy E, Sauvageau E, Genevro J, Guterman LR, Hanel R, et al. Intra/extra-aneurysmal stent placement for management of complex and wide-necked- bifurcation aneurysms: eight cases using the waffle cone technique. Neurosurgery 2006;58:ONS258–62. 10.1227/01.NEU.0000204713.24945.D2 16582648

[9] Lylyk P, Chudyk J, Bleise C, Sahl H, Pérez MA, Henkes H, et al. The pCONus2 neck-bridging device: early clinical experience and immediate angiographic results. World Neurosurg 2018;110:e766–75. 29180089 10.1016/j.wneu.2017.11.097

[10] Sorenson TJ, Iacobucci M, Murad MH, Spelle L, Moret J, Lanzino G. The pCONUS bifurcation aneurysm implants for endovascular treatment of adults with intracranial aneurysms: a systematic review and meta-analysis. Surg Neurol Int 2019 Feb 28;10:24. 31123631 10.4103/sni.sni_297_18 PMC6416758

